# Promoting stakeholder awareness, collaboration, and engagement in One Health surveillance with a serious game

**DOI:** 10.1016/j.onehlt.2025.101257

**Published:** 2025-10-27

**Authors:** Marie-Marie Olive, Saa André Tolno, Brice Lafia, Innocent Ndong Bass, Mamadou Alimou Barry, Mathias Talla Mba, Emmanuel Laury, Helene De Nys, Ahidjo Ayouba, Victor Yacinthe Guigma Wendmisida, François Diaz, Mathieu Bourgarel, Sophie Muset, Marisa Peyre

**Affiliations:** a CIRAD, UMR ASTRE, Montpellier, France; b ASTRE, Université Montpellier, CIRAD, INRAE, Montpellier, France; c MIVEGEC, Université Montpellier, IRD, CNRS, Montpellier, France; d Institut supérieur des sciences et de médecine vétérinaire (ISSMV), Dalaba, Guinea; e World Organisation for Animal Health, Bamako, Mali; f Food and Agricultural Organization of the United Nations, Kinshasa, Democratic Republic of the Congo; g Centre de Recherche sur les Maladies Emergentes et Réémergentes (CREMER), Yaounde, Cameroon; h Office Guinéen des Parcs Nationaux et Réserves de Faune (OGPNRF), Conakry, Guinea; i CIRAD, UMR ASTRE, Harare, Zimbabwe; j TransVIHMI, Université Montpellier, INSERM, IRD, Montpellier, France; k World Organisation for Animal Health, Paris, France; l Centro de Biotecnologia-UEM, Universidade Eduardo Mondlane, Maputo, Mozambique

**Keywords:** Serious game, One Health, Zoonotic diseases, Surveillance system, Rapid response system, Community-based surveillance system

## Abstract

The COVID-19 pandemic and recurrent Mpox and Ebola virus epidemics have revealed a critical need for One Health disease surveillance systems, and especially systems with integrated community-based approaches for early disease detection. Local stakeholders, including community workers in the human, animal, and environmental health sectors, are at the forefront of emerging disease detection and early warnings. However, their involvement in surveillance systems remains limited, even though they play a critical role in event- and community-based surveillance and responses. We developed a collaborative board game, called ALERT, to strengthen stakeholder engagement across all levels of One Health surveillance systems in West and Central Africa. This serious game has generated significant enthusiasm among the 500 people who have tested it in over 100 countries.

## 1 Short communication

The COVID-19 pandemic and recurrent Mpox and Ebola virus epidemics have shown a critical need for One Health disease surveillance systems, and especially systems with integrated community-based approaches for early disease detection [1]. Local stakeholders, including community workers in the human, animal, and environment health sectors, are at the forefront of emerging disease detection and early warnings. However, their involvement in surveillance systems remains limited, even though they play a critical role in event- and community-based surveillance and responses [2,3]. A better understanding of how surveillance systems work, how they could be improved, and the roles stakeholders should play is also needed. Filling these gaps requires innovative approaches to ensure inclusion, training, and sustained engagement of stakeholders.

It is commonly suggested that people remember what they experience better than what they hear when learning [4]. Serious games have emerged as promising tools to enhance collaboration, learning, and stakeholder engagement in various fields, including health [5].

**Fig. 1:**
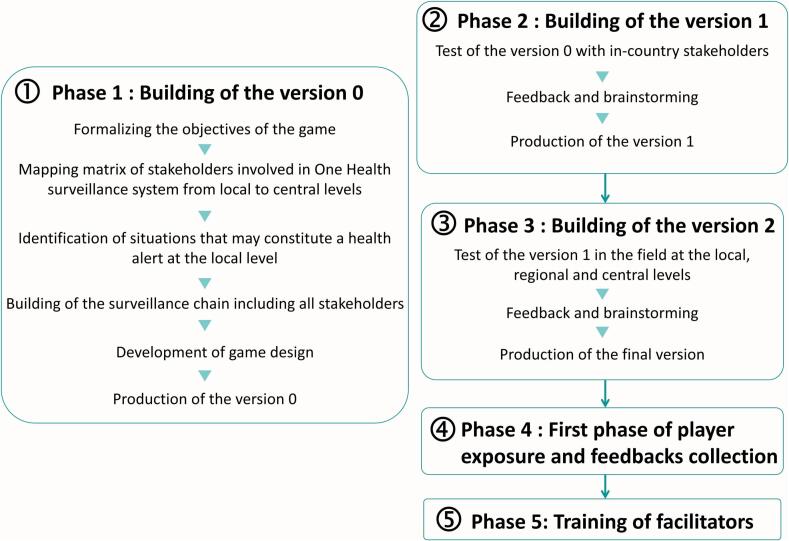
The process to produce and disseminate ALERT.

We developed a collaborative board game, called ALERT, designed to strengthen stakeholder engagement across all levels of One Health surveillance systems in West and Central Africa [6,7]. ALERT was created, within EBO-SURSY project (https://ebo-sursy.woah.org/), using an iterative process involving game development experts (Bioviva, https://www.bioviva.com/en/) and surveillance system specialists from Central and West Africa and France and was tested in the field at the local, regional and central levels (Fig. 1) [6,7]. The game was developed to support One Health surveillance by promoting best practices, highlighting stakeholders' roles and responsibilities when facing health threats, and fostering dialogue among stakeholders.

**Fig. 2:**
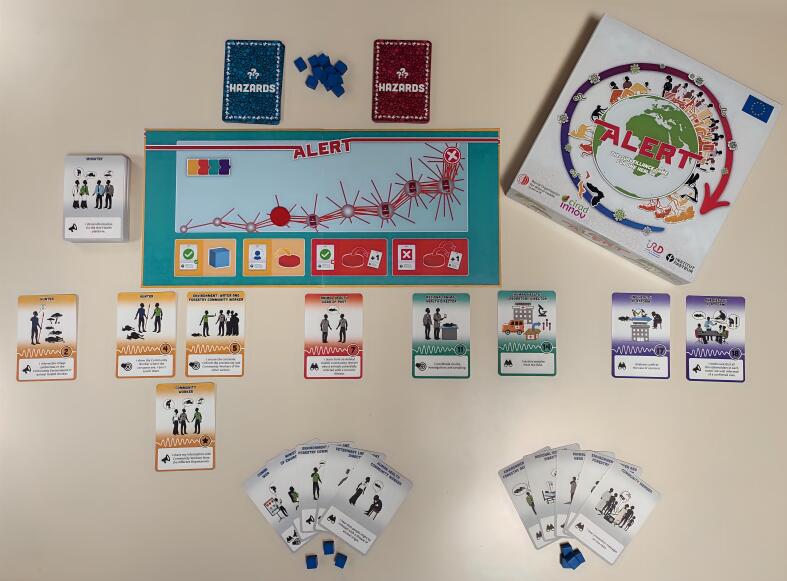
Example of an ALERT game play. Image quality enhanced by artificial intelligence (https://aiimagegenerator.is).

ALERT was developed based on four core learning objectives: (i) to raise awareness about how One Health collaboration functions within surveillance systems, (ii) to raise awareness among stakeholders from the environmental, public, and animal health sectors of what their roles and responsibilities should be within surveillance systems, (iii) to enhance collaboration between stakeholders from local to national levels, and (iv) to increase stakeholder engagement in One Health surveillance systems. The game includes more than 80 stakeholder roles, divided into four levels: local, sub-regional, regional, and national/central (Fig. 2). The game targets adult players. Players collaborate to enhance health information flows and control actions from citizens to central authorities, simulating real-life, event-based surveillance to prevent disease emergence. The ALERT game also raises awareness of the consequences of failing to take action at local level in the spread of an epidemic.

The game always requires the presence of a facilitator who plays a crucial role in guiding game sessions. They are trained over four days to master the game mechanics, understand infectious disease surveillance concepts, and adopt a participatory approach for leading discussions. Since 2022, facilitator trainings have been held in Senegal, Cameroon, Côte d'Ivoire and France. To date, 50 facilitators from Cameroon, Gabon, France, Guinea, Togo, Senegal, Burkina Faso, Côte d'Ivoire, and Madagascar have participated in the deployment of ALERT in various contexts, such as field studies, academic trainings, and scientific symposiums. Demonstration sessions have also been held for approximately 500 people from 104 different countries in Africa, Europe, and Asia. Participants included local health workers, veterinarians, wildlife conservationists, medical doctors, laboratory technicians, civil servants, researchers, staff from international organisations (such as FAO, WOAH, and WHO), and university educators. They showed a strong interest in playing collaboratively and discussing multisectoral strategies to share information and manage disease emergence [6]. The game therefore not only allows for community worker engagement in the surveillance chain but also helps build up the core capacities of national health experts.

A preliminary evaluation of ALERT deployment in the field in Guinea demonstrated its effectiveness in fostering dialogue among stakeholders, improving knowledge of surveillance concepts, and strengthening engagement, particularly among community workers [8]. Local stakeholders also found the game entertaining and useful, and they asked to keep a copy so they could continue playing it outside organised learning sessions. The game's relevance for teaching and discussing surveillance practices has also been recognised by trained academics and One Health professionals. Future plans include expanding facilitator training programmes, monitoring the game's long-term impact on stakeholder knowledge and engagement, and adapting ALERT for European and Southeast Asian contexts.

Training opportunities and knowledge acquisition are the main drivers of success of community-based surveillance systems [2,3]. By providing a collaborative and interactive learning platform, ALERT has the potential to sustainably engage stakeholders, including the local communities in One Health surveillance systems. Innovative tools such as the serious game ALERT are promising ways to address the challenges of inclusion, training, and engagement in One Health surveillance, ultimately contributing to more effective community-based surveillance systems.

## Funding sources

This work was supported by the 10.13039/501100000780European Union under the Agreement FOOD/2016/379–660 for the implementation of the EBO-SURSY Project “Capacity building and surveillance for viral haemorrhagic fevers” (https://rr-africa.oie.int/en/projects/ebo-sursy-en/).

## CRediT authorship contribution statement

**Marie-Marie Olive:** Conceptualization, Formal analysis, Investigation, Methodology, Supervision, Validation, Visualization, Writing – original draft, Writing – review & editing. **Saa André Tolno:** Formal analysis, Investigation, Validation, Writing – review & editing. **Brice Lafia:** Conceptualization, Investigation, Methodology, Project administration, Supervision, Validation, Writing – review & editing. **Innocent Ndong Bass:** Investigation, Validation, Writing – review & editing. **Mamadou Alimou Barry:** Investigation, Validation, Writing – review & editing. **Mathias Talla Mba:** Investigation, Methodology, Validation, Writing – review & editing. **Emmanuel Laury:** Formal analysis, Investigation. **Helene De Nys:** Investigation, Project administration, Supervision, Validation, Writing – review & editing. **Ahidjo Ayouba:** Investigation, Validation, Writing – review & editing. **Victor Yacinthe Guigma Wendmisida:** Investigation, Project administration, Supervision, Validation, Writing – review & editing. **François Diaz:** Conceptualization, Investigation, Methodology, Validation, Writing – review & editing. **Mathieu Bourgarel:** Investigation, Project administration, Supervision, Validation, Writing – review & editing. **Sophie Muset:** Conceptualization, Funding acquisition, Investigation, Methodology, Project administration, Supervision, Visualization, Writing – review & editing. **Marisa Peyre:** Conceptualization, Funding acquisition, Investigation, Methodology, Supervision, Validation, Visualization, Writing – original draft, Writing – review & editing.

## Declaration of competing interest

The authors declare that they have no known competing financial interests or personal relationships that could have appeared to influence the work reported in this paper.

## Data Availability

No data was used for the research described in the article.
